# Prevalence and Genetic Diversity of *Cronobacter* Species Isolated From Four Infant Formula Production Factories in China

**DOI:** 10.3389/fmicb.2019.01938

**Published:** 2019-08-21

**Authors:** Yan Lu, Peng Liu, Changguo Li, Miao Sha, Jingquan Fang, Jingwen Gao, Xiaoxi Xu, Karl R. Matthews

**Affiliations:** ^1^College of Food Science, Northeast Agricultural University, Harbin, China; ^2^Green Food Research Institute of Heilongjiang (National Research Center of Dairy Engineering and Technology), Northeast Agricultural University, Harbin, China; ^3^Food Department, Heilongjiang Administration for Market Regulation, Harbin, China; ^4^Department of Food Science, Rutgers, The State University of New Jersey, New Brunswick, NJ, United States

**Keywords:** *Cronobacter* spp., genetic diversity, infant formula production facilities, multi-locus sequence typing, pulsed-field gel electrophoresis

## Abstract

The aim of this study was to investigate the prevalence and genotypic characteristics of *Cronobacter* isolated from powdered infant formula (PIF) manufacturing facilities and to identify a potential source of contamination. A total of 42 *Cronobacter* isolates (5%) were detected in 835 environmental samples collected during the surveillance study. These isolates included *C*. *sakazakii* (*n* = 37), *C*. *malonaticus* (*n* = 3), and *C. turicensis* (*n* = 2). The isolates were divided into 14 sequence types (STs) by multi-locus sequence typing (MLST) and 21 pulsotypes (PTs) using pulsed-field gel electrophoresis (PFGE). The dominant *C. sakazakii* sequence types were ST3 (*n* = 12) and ST21 (*n* = 10), followed by ST136 (*n* = 6). The major PTs were PT22 (*n* = 12) and PT17 (*n* = 4) based on 100% similarity. Strains isolated from samples collected at the same production facility showed closer phylogenetic relation than those collected from distinct facilities. The result of extensive traceback sampling showed that PIF residues (PIF dust in production areas), fluid beds, drying areas, floors, and soil samples collected adjacent to the production facilities were the primary positive areas for *Cronobacter*. The present study outlines an effective approach to determine prevalence and genetic diversity of *Cronobacter* isolates associated with contamination of PIF.

## Introduction

*Cronobacter* spp. belongs to the family Enterobacteriaceae, which were previously classified as *Enterobacter sakazakii*, consisting of *C. sakazakii*, *C. malonaticus*, *C. turicensis*, *C. muytjensii*, *C. condimenti*, *C*. *universalis*, and *C. dublinensis* (Stephan et al., 2014). *Cronobacter* infections in infants can lead to necrotizing enterocolitis, septicemia, meningitis, and death (FAO/WHO 2004, 2006, 2008). Powdered infant formula (PIF) is a major vehicle for *Cronobacter* infections in neonates (Stephan et al., 2011; Hunter and Bean, 2013). Building on information related to *Cronobacter* prevalence and genetic diversity will prove valuable in conducting risk analyses to identify the potential sources of *Cronobacter* during production of PIF.

A plethora of studies have shown that *Cronobacter* spp. can be isolated from PIF and PIF manufacturing facilities (Iversen et al., 2009; Craven et al., 2010; Fei et al., 2018b). Mullane et al. (2007) profiled *Cronobacter* spp. isolates by pulsed-field gel electrophoresis (PFGE) from a PIF production site and demonstrated the persistence of specific bacterial clones in the production facility. Proudy et al. (2008) highlighted that the environment is the major source of *Cronobacter* contamination of PIF and PIF production facilities. However, the contamination source and transmission routes of *Cronobacter* may differ depending on the production process and country of production. In depth studies focused on sources of *Cronobacter* within production environments are limited; most sampling is conducted on finished products.

In the present study, traceback sampling of the PIF production facility environment was conducted to determine: (1) areas within a production facility and area(s) immediately outside the facility that may harbor *Cronobacter*, (2) identify processing step(s) that represent a greatest risk for introduction of *Cronobacter* into PIF, and (3) determine prevalence and genotypic characterization of *Cronobacter*. A total of 835 environmental samples were collected and 42 *Cronobacter* strains were identified. Assessment of the genetic diversity of *Cronobacter* isolates was conducted using multi-locus sequence typing (MLST) and pulsed-field gel electrophoresis (PFGE) to determine whether single or multiple strains were dominant in a given PIF production facility and to establish potential sources of contamination.

## Materials and Methods

### Sample Collection

A sampling scheme was developed to cover all the potential source sites of contamination within a PIF production facility collectively referred to as environmental samples. A total of 835 samples were collected from four PIF production facilities in China (Table 1). The environmental samples were collected from clean work areas and quasi work areas (including the spray-drying areas along the entire production line), PIF residues (PIF dust that typically settles on the environmental surfaces) on the equipment surfaces and the floor. In addition to samples collected from the indoor production environment, areas outside the production facility were sampled including air conditioning units, water, and soil. The uniforms, hands, and gloves of technicians conducting the sampling were also sampled.

**Table 1:** Occurrence of *Cronobacter* strains of environmental samples in four infant formula production factories in China.

Environment samples		Numbers of samples	Positive^*^	Isolates in factories
Areas	Locations			
Clean^1^	Inside the equipment after the dryer before packaging, packaging areas (packaging materials), tools for direct contact with powder (weighing related or mixed), infant powder residues	358	13 (3.6%)	Factory A: 1
				Factory B: 0
				Factory C: 0
				Factory D: 12
Quasi-clean^2^	External parts of equipment: the surfaces of the spray-drying, equipment and associated processing equipment (fluid beds^4^), floors surrounding the line, infant powder residues, pipeline, platforms	305	23 (7.5%)	Factory A: 6
				Factory B: 8
				Factory C: 0
				Factory D: 9
Common^3^	Raw material warehouse and finished products warehouse	32	0	0
Outside	Air conditioners, water tank, and soils outside	36	5 (13.9%)	Factory A: 5
				Factory B: 0
				Factory C: 0
				Factory D: 0
Staffs	Hands of operating workers, uniforms, surface of gloves, shoes	56	1 (1.8%)	Factory A: 1
				Factory B: 0
				Factory C: 0
				Factory D: 0
Others	Washing machines, drains, vacuum cleaners, trash cans	48	0	0
Total		835	42 (5.0%)	

1 *Clean areas: high cleanliness requirement of operational areas, such as exposure to the packaging of semi-finished product storage, filling and inner packing workshop*.

2 *Quasi-clean areas: a lower cleanliness requirement than clean area, such as pretreatment workshop*.

3 *Common areas: a lower cleanliness requirement than quasi-clean area, such as warehouse*.

4 *Fluid bed: equipment used for dehumidification and to reduce powder temperature*.

* *The locations for C. sakazakii positive in first detection will be tested again around the sampling area to assess the C. sakazakii prevalence, all the detection will be completed in a week*.

A sterile sponge-stick was used to swab the surface of a defined area (100 cm^2^ or 1 m^2^) of the floor, the equipment, and the packaging materials. A sterile swab was used to sample a defined area (20 cm^2^) of irregular surfaces and the entire area of a hand, or inner wall of a PIF can. Sponges and swabs were returned to the laboratory and processed within 4 h. Surfaces were disinfected with ethanol and dried after sampling.

### Isolation and Identification of *Cronobacter* Strains

Processing of samples was conducted as follows: (1) sponge samples were added into 100 ml of sterile buffered peptone water (BPW, LandBridge, China), mixed well, then incubated for 18 ± 2 h at 36°C; (2) swab samples containing 10 ml of sterile BPW were incubated directly for 18 ± 2 h at 36°C; and (3) PIF residue samples were mixed with BPW to yield a tenfold dilution for pre-enrichment (36°C for 18 ± 2 h). After incubation, a 1 ml aliquot of the pre-enrichment culture was transferred into 10 ml of modified lauryl sulfate tryptose (mLST, LandBridge, China)/vancomycin medium and then incubated at 44 ± 0.5°C for 24 ± 2 h. All samples were streaked onto the CHROMagar™ *Cronobacter* spp. agar (CHROMagar, France) and incubated at 37°C for 24 ± 2 h. Presumptive colonies of *Cronobacter* were selected, and streaked onto tryptone soy agar (TSA, LandBridge, China), followed by incubation at 25°C for 44–48 h. After incubation, presumptive *Cronobacter* colonies were selected to confirm their identity using biochemical identification and 16S rRNA gene sequencing (Chen et al., 2010; ISO 22964, 2017).

All the confirmed *Cronobacter* isolates were cryopreserved at −80°C in 40% (v/v) glycerol in water. Prior to analysis of MLST and PFGE, isolates were cultured in BPW at 36°C for 18 ± 2 h, streaked onto TSA and incubated at 36°C for 24 h to isolate a single colony.

### MLST Subtyping of *Cronobacter*

Genomic DNA was extracted using a Tsingke Bacteria DNA kit (TsingKe Biotech Co., Ltd., Beijing, China). Primers for the seven housekeeping genes (*atpD*, *fusA*, *glnS*, *gltB*, *gyrB*, *infB*, and *ppsA*) and PCR was performed according to Joseph et al. (2012) and Cui et al. (2015). The PCR products were sequenced by TsingKe Biotech Co., Ltd. (Beijing, China). The assignments of each allele and sequence types (STs) profile were obtained from the *Cronobacter* MLST open-access database (http://pubmlst.org/Cronobacter/). The phylogenetic relationship of the concatenated sequences (3,036 bp) of the seven housekeeping genes was analyzed using the maximum-likelihood algorithm in MEGA (version 6) with 1,000 bootstrap replicates. A minimum spanning tree was constructed to analyze the relatedness of all isolates using BioNumerics (version 5).

### PFGE Subtyping of *Cronobacter*

*Cronobacter* isolates were analyzed as described in the PulseNet standardized PFGE *Cronobacter* protocol using *XbaI* (TaKaRa, Japan) as the restriction enzyme (Brengi et al., 2012; Yan and Fanning, 2015). DNA fragments were separated by electrophoresis (CHEF Mapper, Bio-Rad Laboratories, Hercules, California, US) through a 1% (w/v) agarose gel (Seakem Gold, Rockland, Maine, US) in 0.56 TBE buffer at 6 V/cm with an initial switch time of 1.8 s and a final switch time of 25 s. Gels were stained in deionized water containing GelRed Nucleic Acid Gel Stain (Biotium, CA, US), and visualized under UV light using a GelDoc XR^+^ system (Bio-Rad laboratories, Hercules, California, US). *XbaI*-digested *Salmonella* Braenderup H 9812 was used as the molecular weight standard. Dendrograms were constructed using Bionumerics software (Version 5.1, Applied-Maths, Belgium), and the cluster analysis was conducted using the DICE coefficient and unweighted pair group method (UPGMA) with arithmetic means, with a 1.2% band position tolerance. When comparing the DNA fingerprint patterns, a cutoff value of 100% similarity was applied.

## Results

### Prevalence of *Cronobacter*

**Figure 1:**
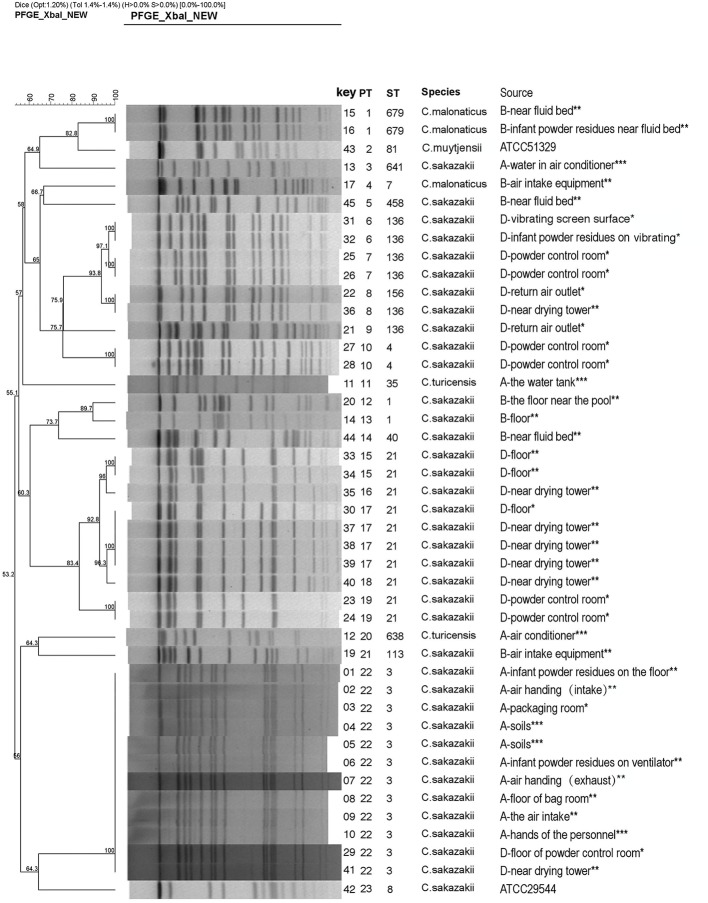
Dendrogram based on XbaI-mediated PFGE profiles of 44 *Cronobacter* spp. The tree was drawn using UPGMA and the Dice coefficient with 1.2% tolerance. Forty-four *Cronobacter* spp. include 42 *Cronobacter* isolates and two reference strains (ATCC 29544 and ATCC 51329). PT, pulsotype; ST, sequence type. **(A, B**, and **D)** indicate the three PIF factories, while no Cronobacter was not isolated in Factory **C**. ^*^The strains from the clean work areas. ^**^The strains from the quasi-clean work areas. ^***^The external environment of the factory.

In total, 835 samples were screened for the presence of *Cronobacter* spp., resulting in 42 isolates, 13 isolates from Factory A, eight from Factory B, zero in Factory C, and 21 from Factory D, respectively. In Factory A, 10 *C. sakazakii* isolates were associated with samples collected from inside and immediately outside the factory, two *C. turicensis* and one *C. sakazakii* were recovered from samples collected from a water tank and air conditioner. In Factory B, eight *Cronobacter* isolates were recovered from “quasi-clean” work areas, adjacent to fluid beds and air intake equipment. Twenty-one *C. sakazakii* isolates; 12 isolated associated with “clean work” areas and nine associated with “quasi-clean” work areas (PIF residues, powder control room, processing line, and the drying tower) in Factory D (Table 1 and Figure 1).

The majority of *Cronobacter* strains were recovered from outside areas in this study (13.9%, 5/36). The “quasi-clean” work areas including the spray-drying areas, and the floor (23 strains) also accounted for large number of strains. In “clean work” areas, positive samples were associated with the PIF control room, return air outlet, and PIF residues (associated with various surfaces) (3.6%, 13/358) (Table 1). In Factories A, B, and D, four positive sites for *Cronobacter* were associated with PIF residues. The remaining positive samples were recovered from outside the production facility and staff. No positive samples were collected from common areas (Figure 1).

### MLST Profiling

**Figure 2:**
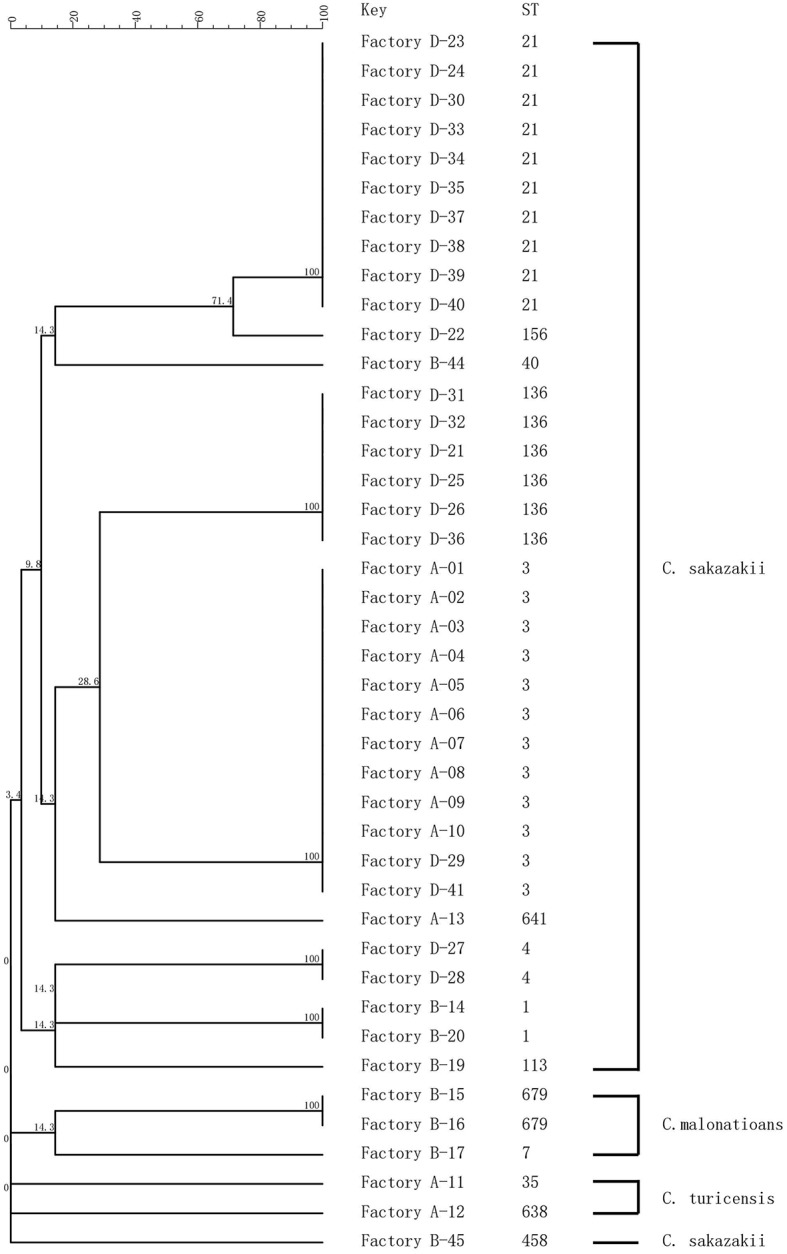
MLST dendrogram of 42 *Cronobacter* strains. Comparison is constructed from concatenated sequences of the seven MLST loci.

Sequence trace files were used to generate a minimum spanning tree (Figure 2). MLST subtyping was performed with all 42 isolates which were clustered into 14 STs, of which 37 isolates were identified as *C. sakazakii*, three isolates as *C. malonaticus*, and two isolates of *C. turicensis*. *C. sakazakii* was the dominant species identified in this study, which included 10 STs, with ST3 (*n* = 12) as the main sequence type, followed by ST21 (*n* = 10) and ST136 (*n* = 6). *C. malonaticus* included two STs (ST679 and ST7), and ST679 was assigned as the new ST in the MLST database. *C. turicensi* included two STs (ST35 and ST638).

### PFGE Analysis

Forty-two *Cronobacter* isolates were divided into 21 pulsotypes (PTs) by using 100% similarity type as the critical threshold. The major PTs were PT22 (*n* = 12) and PT17 (*n* = 4). The isolates identified as *C. sakazakii* (*n* = 37) were typed into 17 PTs. *C. malonaticus* (*n* = 3) and *C. turicensis* (*n* = 2) were typed into two PTs, respectively (Figure 1).

### The Tracing of *Cronobacter* spp. in PIF Factories

In Factory A, 10 *C. sakazakii* isolates exhibited the same type (ST3 and PT22), and they were collected from the hands of technicians as well as the environment inside and outside the factory. In Factory B, five *C. sakazakii* isolates and three *C. malonaticus* isolates were recovered and exhibited six STs and seven PTs. In Factory D, 21 *C. sakazakii* isolates were recovered and included five STs and 11 PTs. Collectively, *Cronobacter* isolates with different sequence types and pulsotypes from the environment exhibited a high diversity. Although the geographic separation of PIF manufacturing facilities included in the study was minimal, each factory harbored strains unique to that factory (Figures 1, 2).

## Discussion

*Cronobacter* spp. are opportunistic foodborne pathogens that can cause severe infections in neonates and infants through the ingestion of contaminated PIF; therefore, they are of global interest to human health (Forsythe et al., 2014; Fei et al., 2018a; Li et al., 2019). Results of this study suggest that should *Cronobacter* be detected in clean area or quasi-clean areas, measures must be immediately implemented to prevent contamination of the end products’ subsequent greater contamination of the processing environment (Pei et al., 2019). In manufacturing, *Cronobacter* may contaminate the processing line and product, since the current technology cannot completely eliminate the pathogen from the manufacturing environment (Codex, 2008; Fei et al., 2015). The study of multiple PIF plants is necessary and justified since surveillance of a single PIF plant would likely fail to provide sufficient evidence for the occurrence of *Cronobacter*. Therefore, in the present study, understanding areas within and external to a PIF production facility that may harbor *Cronobacter* in four PIF factories will aid in developing risk assessments and provide guidance to manufacturers when reviewing environmental monitoring plans.

Focusing on Factory A, ST3 was the predominant strain associated with samples collected from inside and outside of the Factory. *C. sakazakii* PT22 showed that ST3 isolates were identical. Collectively, these results demonstrate that *C. sakazakii* ST3 persistently and extensively contaminated Factory A. Moreover, ST3 exhibited the same type as soil associated isolates suggesting that soil may be the source of the *Cronobacter* contamination in Factory A. Bioaerosols are easily translocated by wind and air currents from one ecosystem to another, facilitating the spread of potentially pathogenic organisms (Wijnand et al., 2012). The bacteria may be brought into the factory through dust particles or condensation droplets, on worker shoes and clothes (Kucerova et al., 2011; Brandl et al., 2014). Dust particles in the air of a manufacturing plant can be a vector of *Cronobacter* dispersal, and the highest particle loads were observed in areas where filling, bagging, and the final packaging of PIF occurs (Brandl et al., 2005; Mullane et al., 2008). To reduce *Cronobacter* contamination in PIF manufacturing facilities, methods that reduce the number of dust particles in the air and treatment of PIF residues may be considered.

In Factory B, one *C. sakazakii* and one *C. malonaticus* were isolated from the air intake equipment, which is likely an essential portal for the transfer of bacteria from the outside environment to the inside of a facility. However, neither was isolated from the inside of Factory B. Two identical *C. malonaticus* (ST679/ PT1) and two *C. sakazakii* were isolated from near the fluid bed, but the two *C. sakazakii* exhibited different sequence types and pulsotypes. *C. malonaticus* strains were not persistent inside the plant, and only isolated in Factory B. Collectively, this suggests that there were multiple sources by which *Cronobacter* was introduced into Factory B. Results also indicated inadequate cleaning and sanitizing of the fluid bed and the floor in Factory B.

In Factory D, the isolate from the return air outlet was identical to the dominant isolates (ST136), suggesting that *C. sakazakii* inside the production facility contaminated the air return outlet by virtue of air flow and PIF transmission (Figure 1). During PIF production, particularly in the spray-drying and packing areas, once the air is contaminated with *Cronobacter* spp., the risk of final product contamination increases (Sonbol et al., 2013).

The best environmental controls for *Cronobacter* of all the factories were perhaps associated with Factory C in which no samples were positive for the pathogen. Factory C used a drying processing method which differs from the wet-mix processing method used by Factories A, B, and D. Perhaps implementation of more effective manufacturing procedures and sanitation programs in conjunction with the reduction of water in the dry-mix process may have contributed to the control of *Cronobacter* by Factory C. Based on the results of this study, more research is needed on the effects of processing technologies to control contamination of PIF by *Cronobacter* spp. PIF processors should implement practices that control the spread of *Cronobacter*, including keeping the air humidity low, reducing formation and spread of dust particles, timely removal of PIF residues, and regular cleaning and sanitizing of production equipment (Codex, 2008; Brandl et al., 2014).

Importantly, sites of contamination in the processing environment and routes of transmission were necessary to aid in risk analysis. Craven et al. (2010) investigated *Cronobacter* contamination in five Australian milk powder plants using PFGE. They suggest that *Cronobacter* strains are spread by air movement, milk powder dust particles, and personnel moving through the production environment. Pei et al. (2019) identified 10 isolates from six sample areas including conveyer belts, floor, walls, water powder around the product line, and vacuum cleaners. *Cronobacter* prevalence was the highest in samples collected from vacuum cleaners (28.0% positive samples). However, in the present study, no isolations were recovered from the vacuum cleaners. Rather, the extensive traceback sampling showed that PIF residues, fluid beds, drying areas, floors, and soil samples were the major contamination sites, which represent the greatest risk for introduction of *Cronobacter* during the production of PIF.

The isolates from samples collected at the same production facility showed closer phylogenetic relation than those collected from distinct facilities. The only exception was ST3, which was isolated from Factories A and D (Figure 2). The main purpose of the present study was to investigate the prevalence and potential contamination source of *Cronobacter* linked to PIF. The study was not designed to address how given strains may contaminate several factories. Consequently, a potential perspective future study could focus on the gene sequence of ST3 strains and the potential source of multiple factory contaminations.

Infant powder formula residues recovered from the production environment were more likely to harbor *C. sakazakii*. Although PIF is a low water activity product, *Cronobacter* spp. can survive in PIF for extended periods of time. Therefore, PIF should be included in environmental monitoring programs for *Cronobacter*. Pei et al. (2019) reported that the occurrence of *Cronobacter* is mainly associated with powder clumps on the vibrating screen in the processing environment. Those results are consistent with the present study where four positive sites for *Cronobacter* were associated with PIF residues.

In the present study, multiple locations within a PIF manufacturing facility were positive for the pathogen. Notably, *C. sakazakii* ST3, ST21, and ST136 were the dominant ST in this study. They are widely isolated from food (mainly PIF) and the environment according to PubMLST database, but no report was associated with human infection in China. Some pathogenic STs (e.g., ST1, ST7 from Factory B and ST4 from Factory D) were also identified in this study. They are commonly related to clinical cases based on MLST analysis. For example, *C. sakazakii* ST4 is linked to cases of meningitis (Hariri et al., 2013) and *C. malonaticus* ST7 is associated with adult infections, though the source was not identified (Joseph et al., 2012). Therefore, it is critical to investigate the *Cronobacter* distribution in the environment, which will help in understanding how the final product was contaminated and where the pathogens came from. Comparison of MLST and PFGE, as performed in this study, are useful for PIF manufactures to understand the diversity and characteristics of *Cronobacter.*

This study has improved our understanding of the prevalence and genetic diversity of *Cronobacter* spp. isolated from the production environment of PIF manufacturing facilities in China. Prevention efforts at manufacturing facilities must be multi-faceted and should include environmental sampling, and best practices supported by government regulatory agencies. Public education programs supported by health-care providers that emphasize safe handling practices of PIF will contribute to ensuring consumer safety. PIF manufacturers can utilize the results as a basis to review their existing monitoring program as part of a food safety plan and to make appropriate revisions to control PIF contamination.

## Data Availability

The raw data supporting the conclusions of this manuscript will be made available by the authors, without undue reservation, to any qualified researcher.

## Author Contributions

XX and YL conceived and designed the experiments. YL, PL, MS, and JF performed the experiments. YL, XX, and CL generated and analyzed the data. YL, JG, and KM wrote the paper.

### Conflict of Interest Statement

The authors declare that the research was conducted in the absence of any commercial or financial relationships that could be construed as a potential conflict of interest.
